# Urgency Promotes Affective Disengagement: Effects From Bivalent Cues on Preference Formation for Abstract Images

**DOI:** 10.3389/fpsyg.2020.01404

**Published:** 2020-06-23

**Authors:** Ji Xu, Noha Mohsen Zommara, Kajornvut Ounjai, Muneyoshi Takahashi, Shunsuke Kobayashi, Tetsuya Matsuda, Johan Lauwereyns

**Affiliations:** ^1^Graduate School of Systems Life Sciences, Kyushu University, Fukuoka, Japan; ^2^Faculty of Engineering, King Mongkut’s University of Technology Thonburi, Bangkok, Thailand; ^3^Brain Science Institute, Tamagawa University, Tokyo, Japan; ^4^Department of Neurology, Teikyo University, Tokyo, Japan; ^5^Faculty of Arts and Science, Kyushu University, Fukuoka, Japan

**Keywords:** preference formation, cueing, gaze cascade, affective association, urgency, gradual commitment versus information integration

## Abstract

Although previous research has characterized the important role for spatial and affective pre-cues in the control of visual attention, less is known about the impact of pre-cues on preference formation. In preference formation, the gaze cascade phenomenon suggests that the gaze serves both to enhance and express “liking” during value-based decision-making. This phenomenon has been interpreted as a type of Pavlovian approach toward preferred objects. Decision-making here reflects a process of gradual commitment in which the gaze functions as a precursor to choice; by this account, overt attention produces a necessarily positive, additive effect on the value of the attended object. The implication is that drawing attention to an object should initiate, and therefore promote, preference formation for that object. Alternatively, information-integration models of attention propose that attention produces a multiplicative effect on the value of the attended object, implying that negative information can impede preference formation. To pitch the gradual-commitment hypothesis against the information-integration hypothesis, we conducted four experiments that combined the spatial-cueing paradigm with a value-based choice paradigm. In each trial in all experiments, subjects were presented with an irrelevant, peripheral pre-cue for a duration of 500 ms, followed by a 500 ms blank, and then a pair of abstract images (one at the pre-cued position; one in the opposite hemifield). The subjects were asked to choose their preferred abstract image by pressing the corresponding button. We manipulated the type of pre-cues (images of faces versus foods; with varying affective associations) and the time constraints (a deadline of 1,500 ms versus self-paced). Overall, the choice data showed a clear pattern of influence from the pre-cues, such that, given a deadline, abstract images were chosen less often if they had been preceded by a pre-cue with a negative affective association (both for face and food images). Analyses of the gaze data showed the emergence of significant gaze biases in line with the subjects’ choices. Taken together, the data pattern provided support for the information-integration hypothesis, particularly under urgency. When tasked with a speeded preference choice, subjects showed affective disengagement following pre-cues that carried a negative association.

## Introduction

Preference formation can be defined as the process of making evaluative judgments on the basis of perceived attractiveness. In general, evaluative decision-making is thought to be subjective, dependent on an individual’s experience and memory as well as the current environment (Stevens, 2008). An influential study on preference formation conducted by Shimojo and his colleagues found a systematic relationship between gaze and preference, called the “gaze cascade,” indicating a positive effect of overt visual attention on preference formation (Shimojo et al., 2003). In this eye-tracking study, pairs of images (faces in some conditions; abstract art images in other conditions) were presented on the screen and subjects were required to choose the more attractive one. It was found that subjects’ gaze tends to gradually orient toward their final choice, starting from more than half a second before the actual decision. In other words, people appear to commit to their preferred choice by spending more time looking at it. As an explanation for the effect, Shimojo and colleagues suggested that the orienting behavior itself not only expresses the likelihood of choice, but also generates the preference for the item being gazed at, resulting in an enhancement of preference.

The hypothesis of a direct connection between human gaze and preference formation can be characterized as a form of “Pavlovian approach,” by which the behavioral orienting serves in effect as a precursor to the choice (Lauwereyns, 2012). Here, we will refer to this proposal as the “gradual-commitment hypothesis.” The evidence of a tight connection between looking and liking was shown to be highly reproducible (including in our own laboratory) in a wide variety of conditions (Simion and Shimojo, 2006, 2007; Glaholt and Reingold, 2009; Schotter et al., 2010; Bird et al., 2012; Morii and Sakagami, 2015; Zommara et al., 2018). However, several lines of research suggest that the relationship between orienting behavior and preference formation may be complex, as a function of the nature of the stimuli being processed. Even within the literature on Pavlovian conditioning, evidence indicates that parallel learning systems may be at work, leading to dissociable effects for gaze direction and affective evaluation (Pool et al., 2019). More generally, the orienting behavior in preference formation might involve covert mechanisms of attention in addition to the overt attention, raising the possibility of different types of information integration that do not necessarily equate attention with enhancement of preference formation.

The issue can be specified more formally via an accumulator model of preference formation developed by Krajbich et al. (2010). The hypothesis of a direct connection between gaze and preference formation amounts to an additive accumulator model, by which gaze duration necessarily leads to a positive increase in the value of the attended object. Conversely, as indeed anticipated by Krajbich and colleagues, it seems reasonable to hypothesize a multiplicative relationship between attention and value, with increased gain as a function of gaze duration. The implication here is that attention to negatively valued stimuli should hyperpolarize the preference formation in the negative direction, making the attended object less likely to be chosen. Recent work by Gluth et al. (2018, 2020) demonstrates the importance of distinguishing between additive and multiplicative accumulator models of preference formation.

The hypothesis of a multiplicative relationship between attention and value is consistent with classic work on the “feature-integration theory of attention” (Treisman and Gelade, 1980) proposing that attention serves a binding function in information processing. Although this theory was proposed with respect to the integration of primitive visual features, later work has expanded the notion of an integrator function of attention to other types of information, including affective features as well as complex or abstract semantic features (e.g., Raymond et al., 2005; Li et al., 2016; Kunar et al., 2017; Scharinger et al., 2017). The corollary of this notion is that attention to an object might lead to either an increase or a decrease in the value of the attended object, depending on the nature of the information being integrated. Thus, drawing attention to negative information with respect to an object would lead to a gradual devaluation of the attended object, whereas positive information would promote the valuation. We will refer to this hypothesis as “the information-integration hypothesis.”

Furthermore, in the area of research on covert visual attention, Posner (1980) introduced the role of pre-cueing in the orienting of attention using the spatial cueing paradigm (for comprehensive reviews of related work, see Carrasco, 2011; Atkinson et al., 2018). The paradigm was based on a perceptual decision-making task in which a peripheral pre-cue may indicate the location of a subsequent target. Depending on the length of the time interval in between the onset of the cue and the target, the effect of the pre-cueing on response could be either facilitative or inhibitory. For instance, with a short time interval less than 300 ms, subjects tend to react faster and more accurately to the subsequent target at the cued location (i.e., same location as the cue) than at the uncued location (i.e., opposite location of the cue). Yet, if the interval extends longer than 300 ms, the effect from the peripheral pre-cue would turn to inhibition (i.e., the response to the cued targets becomes slower and less accurate; see Posner and Cohen, 1984; Handy et al., 1999). This inhibitory mechanism has been defined as “inhibition of return” (IOR). According to previous studies, these two different cueing effects are due to the orienting of attention. In the former, a cue attracts the attention and thus leads to an advantage in processing information at the same position; in the latter, with a longer interval, the attention attracted by the cue would be withdrawn from the original attended location (i.e., cued location); as a result, the detection (or the re-orienting behavior) for the cued target requires an additional process and extra effort to return to the previous attended location, leading to a slower reaction to the cued target (Tipper et al., 1994; Pratt et al., 1997; Klein, 1988, 2000). All in all, Posner’s cueing paradigm provided a very effective method of recasting attentional allocation, giving rise to an expansive set of follow-up studies, often using pre-cues that carried semantic or affective associations (e.g., Mathews et al., 1997; Vuilleumier and Schwartz, 2001; Fox et al., 2002; Taylor and Therrien, 2005; Theeuwes and Van der Stigchel, 2006; Stoyanova et al., 2007; Weaver et al., 2012; Armaghani et al., 2014; Denefrio et al., 2017). However, these studies have focused on the process of perceptual decision-making (i.e., detecting or recognizing the targets); to our knowledge, there has been no work adopting Posner’s paradigm to study evaluative decision-making and gaze cascades.

To resolve the unclear relationship between attention and preference formation, we performed a series of experiments combining the cueing paradigm with an evaluative decision-making task, while measuring the subjects’ manual responses and eye movements. Subjects were presented in each trial with a pre-cue, followed by a pair of abstract art images. While being allowed to move their eyes, as in the gaze-cascade paradigm, the subjects had to select their preferred image by pressing a button. Based on a pilot study to establish appropriate experimental procedures and required statistical power (Xu et al., 2016), the timing of the spatial pre-cues was set to elicit IOR: 500 ms pre-cue presentation and 1 s stimulus-onset asynchrony between the pre-cue display and the target display. Here, IOR would imply more attention for the uncued target, and therefore a higher likelihood of choosing the uncued target, as compared to the cued target.

Our present study consisted of four experiments. In the first two experiments, we used images of faces expressing different emotions (happy, neutral, disgust) as pre-cues under two different time constraints: either a deadline of 1,500 ms or no deadline. In the next two experiments, we used images of foods associated with different affective values (either appetitive or aversive) as pre-cues, again with either a deadline of 1,500 ms or no deadline.

For the targets of the preference tasks, we opted in all experiments to use the abstract, unfamiliar shapes (“Fourier descriptors”) employed also by Shimojo et al. (2003) in their original gaze cascade study. We chose Fourier descriptors, rather than faces, as targets to avoid non-affective, category-to-category priming effects from the faces used as pre-cues.

Following the “information-integration” hypothesis, it should be expected that positive cues would increase the value of cued objects (and thereby counteract the IOR), whereas negative cues would decrease the value of the cued objects (and thereby further exacerbate the IOR). That is, the valence of the pre-cue should influence the preference formation. In contrast, according to the “gradual-commitment” hypothesis, any influence from pre-cues on the control of overt attention should always promote the preference formation for the item being gazed at, independent of the valence of the pre-cue.

As for the different time constraints (i.e., with or without deadline), we aimed to examine whether urgency modulates the effects of pre-cueing. A number of studies have established that urgency can have a critical impact in various types of decision-making (Rastegary and Landy, 1993; Waller et al., 2001; Suri and Monroe, 2003). Reddi and Carpenter (2000) showed that urgency strengthens the impact of prior bias on perceptual decision-making. Similarly, we anticipated that urgency could strengthen the impact of pre-cues on evaluative decision-making. Thus, regardless of whether pre-cues would promote or impede preference formation, their influence should be larger with a deadline than without a deadline.

## Materials and Methods

### Participants

All subjects were recruited from Kyushu University. In the Deadline and Self-paced Face-cueing experiments, there were in total 28 subjects (22 males and 6 females; with a mean age of 23.0 ± 1.67 years old, 2 left-handed). In the Deadline Food-cueing experiment, there were 32 subjects (16 males and 16 females; with a mean age of 21.1 ± 2.59 years old, 2 left-handed). Another 32 subjects participated in the Self-paced Food-cueing experiment (11 males and 21 females; with a mean age of 21.7 ± 1.87 years old, 1 left-handed). All subjects were naïve to the purpose of the experiment and had normal or corrected to normal vision. The study was conducted in accordance with the ethical principles of Kyushu University and approved by the Human Ethics Committee of the Faculty of Arts and Science. Each subject received either course credit or monetary compensation of 1,000 yen for their participation. Written informed consent was obtained before the experiment.

### Apparatus

All visual stimuli were presented on a 23.8-inch full high definition flat-panel-monitor, with a display resolution of 1920 × 1080 pixels. To minimize the head movement by subjects, a chin-rest with a forehead-support was used. The monitor screen was set approximately 62 cm from the chin-rest. Subjects used a keyboard to give manual responses, and their eye movements were recorded by Eye Tribe, an eye-tracking device at 60 Hz sampling rate (The Eye Tribe Aps, Denmark); a system with sufficient reliability for present purposes (Ooms et al., 2015; Wolf et al., 2018; Zommara et al., 2018). To prevent the heat build-up of Eye Tribe, a small universal serial bus (USB) fan was used at the same time.

To record the subjects’ eye movements via Eye Tribe, the subjects were asked to focus and follow a dot on the screen for a 16-point calibration (Ooms et al., 2015). After the calibration, the gaze coordinates were calculated through Eye Tribe with an average accuracy of around 0.5°–1° of visual angle. All events and recordings were controlled through code written in Psychopy (version 1.84.2) (Peirce, 2009; Peirce et al., 2019), including the PyTribe library.

### Stimuli

In the Deadline and Self-paced Face-cueing experiments, four different stimuli were used as pre-cues: a white dot (serving as a control) and three different human facial expressions, classified as neutral, happy and disgusted. The face stimuli were selected from the online NimStim Face Stimulus Set (Tottenham et al., 2009). Based on an online questionnaire prior to conducting the experiment (*n* = 55), we selected the images that were voted as most exemplary for the three facial expressions of neutral, happy and disgusted. All cue stimuli were fixed at 200 × 257 pixels, at a distance of 200 pixels from the fixation cross. The target stimuli consisted of a set of 320 computer-generated geometric figures, including 160 symmetrical figures and 160 asymmetrical figures. The set of figures was drawn by a Fourier Descriptor algorithm (Sakai and Miyashita, 1991; Shimojo et al., 2003). All figures were paired with the same category (i.e., two symmetrical or asymmetrical figures paired together), and in total 320 pairs were made for the two experiments (i.e., 160 for the Deadline experiment and the other 160 for the Self-paced experiment). With regard to the pairing, each figure was used two times for pairing with two different figures. By this manipulation, we are able to have 320 unique pairs from the set of 320 geometric figures, even though a single figure would be exposed twice to each subject. All target stimuli were fixed at 480 × 360 pixels, at a distance of 300 pixels from the fixation cross.

In the Deadline and Self-paced Food-cueing experiments, three types of stimuli were used as pre-cues: a white dot (serving as a control) and two different categories of food images, classified as appetitive food cues and aversive food cues. The food images were selected from the database used in another study done in our lab (Ounjai et al., 2018). The database consisted of the FoodCast research image database (FRIDs) (Foroni et al., 2013) with additional non-copyrighted images from the internet (Ounjai et al., 2018). Based on the evaluation scores from our previous study, 160 images were selected as food cues for this experiment: 80 images with the highest evaluation scores were used as appetitive food cues and 80 images with the lowest evaluation scores were aversive food cues. All cue stimuli were fixed at 350 × 350 pixels, at a distance of 257 pixels from the fixation cross. The target stimuli were selected from the same database of face-cueing experiment, but only 240 geometric figures (120 symmetrical and 120 asymmetrical) were used. Similar to the face-cueing experiment, each figure was paired with two different figures to compose 240 unique pairs for experiment. This 240 unique pairs were used in both Deadline and Self-paced Food-cueing experiment. All target stimuli were fixed at 480 × 360 pixels, with the distance of 300 pixels from the fixation cross.

### Procedures

**FIGURE 1:**
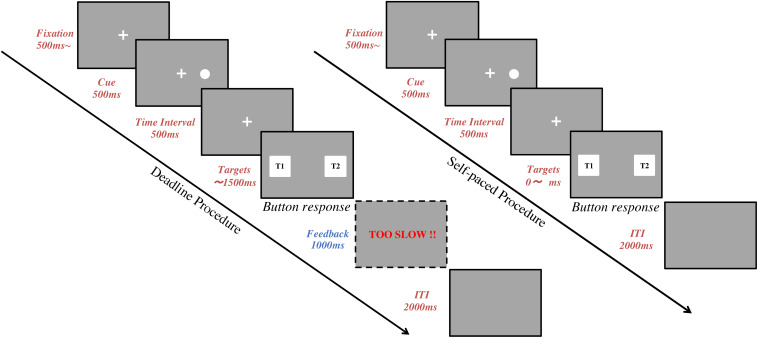
Task procedure. The flow chart represents a trial sequence in the deadline experiments (left) and the self-paced experiments (right). Following fixation, a spatial cue was shown for 500 ms. Then, there was a 500 ms time interval. Finally, two target images were presented, one on the left and one on the right side of the screen, until the subject pressed a response button. In deadline experiments, a feedback screen was shown for 1,000 ms after the target images if the subject failed to give an answer within 1,500 ms. In all experiments, the inter-trial interval (ITI) was 2,000 ms.

In the Deadline Face-cueing experiment (DLFC), there were 4 consecutive blocks of 40 trials. Before starting each block, subjects were asked to fix their head on the chin-rest for the calibration (Ooms et al., 2015). After the subject’s eye positions were calibrated, the subject was instructed to keep their posture without any big movement until the end of the trials. Short breaks were allowed between the 4 blocks. Figure 1 shows the sequence in each trial. A trial started with the presentation of a fixation cross at the center of the screen for at least 500 ms. Subjects were asked to gaze at the fixation until a cue appears. The subjects’ gaze was recorded online and checked in real-time to confirm whether the subject was looking at the fixation cross. After the fixation, a spatial cue for 500 ms would appear on either the left or right side of the fixation. This was followed by a 500 ms time-interval after the cue. Finally, a pair of target images was presented, one on the left and one on the right side of the screen. Subjects were asked to choose the image they preferred within 1.5 s by using their index fingers to press either the left or the right button on the keyboard. The target images disappeared once the subject pressed the button to indicate their response, or when the time reached 1.5 s. A warning message (“TOO SLOW”) was given as feedback for 1 s if the subject failed to give a response before the deadline. A blank screen for 2 s was set for the inter-trial interval (ITI) between each trial.

In the Self-paced Face-cueing experiment (SPFC), the procedures were the same as in DLFC except for the time constraint for responding. In SPFC, the subjects were instructed to compare the two target images carefully and give their response without any time pressure. Since there was no deadline, there were never any warning messages for being too slow in SPFC.

Both DLFC and SPFC had in total 160 trials, consisting of 20 repetitions of 8 conditions, with 4 levels of cue type (Dot, Neutral face, Happy face, Disgusted face), and 2 levels of spatial cueing position (either on the left or on the right side of fixation). The order of trials was pseudo-randomized to ensure that each block of 40 trials contained 5 repetitions of each condition. Before the experimental task, a training session of 20 trials was set for participants to practice. Since DLFC and SPFC were conducted with the same subjects, we counterbalanced the order of the experiments across subjects (i.e., 14 subjects started with the deadline experiment and the other 14 subjects started with the self-paced experiment).

In the Deadline Food-cueing experiment (DLFD), the experimental flow and trial sequence were the same as in DLFC: Subjects were required to select their preferred image within a deadline of 1.5 s. The experiment had in total 240 trials, consisting of 4 consecutive blocks of 60 trials. There were 40 repetitions of 6 conditions, with 3 levels of cue type (Dot, Appetitive food, Aversive food), and 2 levels of spatial cueing position (either on the left or on the right side of fixation). The order of trials was pseudo-randomized to ensure that each block of 60 trials contained 10 repetitions of each condition. Before the experimental task, a training session of 20 trials was set for participants to practice.

In the Self-paced Food-cueing experiment (SPFD), the procedure was the same as in DLFD except that there was no time limit for the response.

## Results

In the deadline experiments, all trials in which subjects failed to give a button-press response within 1.5 s were excluded from the analysis. For this reason, there was a total of 1.5% of all trials rejected for both behavioral and gaze analysis in the DLFC, and 1.6% in the Deadline Food-cueing experiment. In addition, Bonferroni correction was used to set the alpha level at 0.05 for the entire data set in each experiment, in all statistical analyses.

### Manual Response Analysis

#### Probability of Choice

The probability of choosing the uncued target was calculated in all trials for each type of cue, by dividing the number of trials in which the uncued image was chosen by the total number of trials. This index ranged from 0 to 1; the higher, the more choices for uncued images, in line with the hypothesis of IOR. To test whether there was a significant bias in the target choice, for each type of cue we compared the probability of choosing the uncued target against the chance level (0.5) by a two-tailed one-sample *t*-test.

##### Deadline face-cueing experiment (DLFC)

**FIGURE 2:**
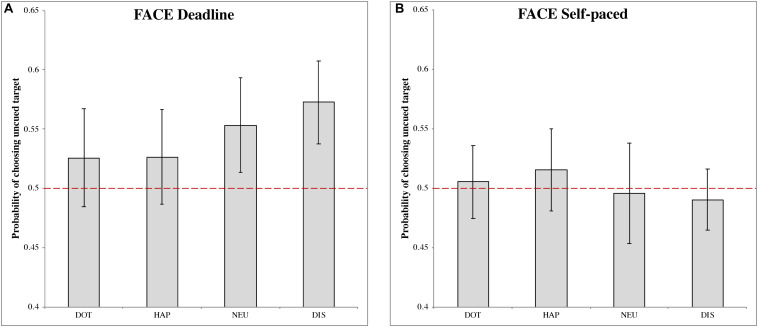
The probability of choosing the uncued target in the face-cueing experiments. **(A,B)** Represent the results of the deadline and the self-paced experiment respectively. Both panels show the data for different types of cue: dot (DOT), happy face (HAP), neutral face (NEU) and disgusted face (DIS). The red dashed line is the chance level (0.5) of selection. The error bars represent the 95% confidence interval around the mean in each condition.

In the DLFC (Figure 2A), we found a significant choice bias following the disgusted face pre-cue (M_DIS_ = 0.573, 95% CI = [0.538, 0.607], *t*(27) = 4.087, *p* < 0.001, Cohen’s *d* = 0.773). This result indicated a higher choice probability of uncued images, under urgency, when the cue is a disgusted face. There were no significant choice biases following the other pre-cues: dot (M_DOT_ = 0.526, 95% CI = [0.483, 0.567], *t*(27) = 1.216, *p* = 0.234, Cohen’s *d* = 0.230); happy face (M_HAP_ = 0.526, 95% CI = [0.486,0.566], *t*(27) = 1.294, *p* = 0.207, Cohen’s *d* = 0.245); and neutral face (M_NEU_ = 0.553, 95% CI = [0.513, 0.593], *t*(27) = 2.613, *p* = 0.014, Cohen’s *d* = 0.494).

##### Self-paced face-cueing experiment (SPFC)

In the SPFC (Figure 2B), no significant choices biases were obtained for any of the pre-cues: dot (M_DOT_ = 0.505, 95% CI = [0.475, 0.536], *t*(27) = 0.342, *p* = 0.735, Cohen’s *d* = 0.065); happy face (M_HAP_ = 0.515, 95% CI = [0.481, 0.550], *t*(27) = 0.862, *p* = 0.396, Cohen’s *d* = 0.163); neutral face (M_NEU_ = 0.496, 95% CI = [0.453, 0.538], *t*(27) = −0.207, *p* = 0.837, Cohen’s *d* = −0.039); disgusted face (M_DIS_ = 0.490, 95% CI = [0.464, 0.516], *t*(27) = −0.744, *p* = 0.463, Cohen’s *d* = −0.141).

##### Deadline food-cueing experiment (DLFD)

**FIGURE 3:**
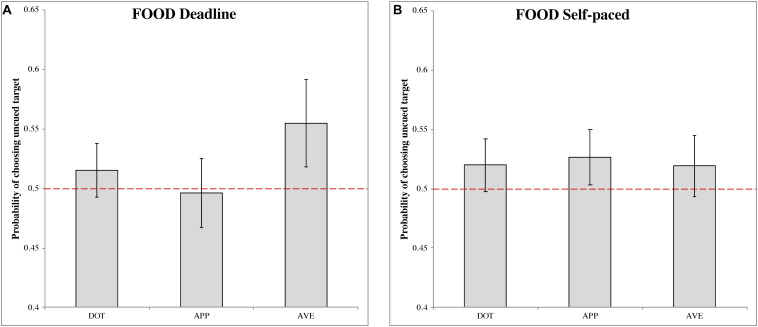
The probability of choosing the uncued target in the food-cueing experiments. **(A,B)** Represent the results of the deadline and the self-paced experiment respectively. Both panels show the data for different types of cue: dot (DOT), appetitive food images (APP), and aversive food images (AVE). The red dashed line is the chance level (0.5) of selection. The error bars represent the 95% confidence interval around the mean in each condition.

In the DLFD (Figure 3A), there was a significant choice bias following aversive food pre-cues (M_AVE_ = 0.555, 95% CI = [0.518, 0.592], *t*(31) = 2.903, *p* < 0.01, Cohen’s *d* = 0.513). This result indicated a higher choice probability of uncued images, under urgency, when the cue is a disgusting food image. There were no significant choice biases following the other pre-cues: dot (M_DOT_ = 0.515, 95% CI = [0.493, 0.538], *t*(31) = 1.340, *p* = 0.190, Cohen’s *d* = 0.237); and appetitive food (M_APP_ = 0.497, 95% CI = [0.468, 0.525], *t*(31) = −0.241, *p* = 0.811, Cohen’s *d* = −0.043).

##### Self-paced food-cueing experiment (SPFD)

In the SPFD (Figure 3B), no significant choices biases were obtained for any of the pre-cues: dot (MDOT = 0.520, 95% CI = [0.498, 0.542], t(31) = 1.749, p = 0.090, Cohen’s d = 0.309); appetitive food (MAPP = 0.527, 95% CI = [0.503, 0.550], t(31) = 2.247, p = 0.032, Cohen’s d = 0.397); and aversive food (MAVE = 0.519, 95% CI = [0.494, 0.545], t(31) = 1.466, p = 0.153, Cohen’s d = 0.259). Note that, for appetitive food, the Bonferroni correction produced a non-significant result, although in this case the target value of 0.5 was just outside the 95% CI.

#### Response Time

The response time (RT) was measured from the onset of the target screen until the button was pressed by the subject. The results are reported as averages from all trials as a function of the choice, either for a cued image (“Cued”) or for an uncued image (“Uncued”). To investigate if the RT changed across different conditions, we conducted a two-way repeated measures ANOVA with the within-subjects factors cue type and choice type.

##### Deadline face-cueing experiment (DLFC)

**FIGURE 4:**
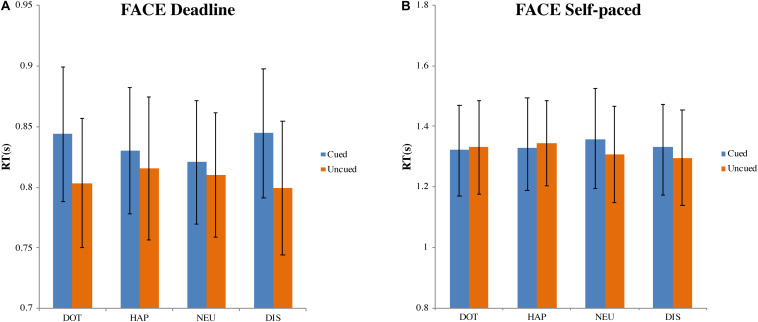
Mean response time and standard errors in the face-cueing experiments. **(A,B)** Represent the results of the deadline and the self-paced experiment respectively. Both panels show the data for different types of cue: dot (DOT), happy face (HAP), neutral face (NEU), and disgusted face (DIS). For each type of cue, the blue bars pertain to the response times when choosing cued targets (Cued); the orange bars pertain to the response times when choosing uncued targets (Uncued). The error bars represent the 95% confidence interval around the mean in each condition.

In the DLFC (Figure 4A), there was a significant main effect of choice (cued versus uncued), *F*(1, 27) = 7.808, MSE = 0.005, *p* < 0.01, η*_*p*_*^2^ = 0.224. However, there was no influence of the cue type, *F*(3, 81) = 0.587, MSE = 0.001, *p* = 0.625, η*_*p*_*^2^ = 0.021; nor was there an interaction between choice and cue type, *F*(3, 81) = 2.085, MSE = 0.002, *p* = 0.109, η*_*p*_*^2^ = 0.072. Compared with the mean of RT for choosing cued images (M_CUED_ = 0.835, 95% CI = [0.782, 0.887]), the RT for choosing uncued images was faster (M_UNCUED_ = 0.807, 95% CI = [0.753, 0.861]).

##### Self-paced face-cueing experiment (SPFC)

In the SPFC (Figure 4B), there was no significant effect of the type of choice, F(1, 27) = 0.523, MSE = 0.025, p = 0.476, ηp^2^ = 0.019, nor of the type of cue, F(3, 81) = 0.422, MSE = 0.013, p = 0.738, ηp2 = 0.015. There was also no interaction between choice and cue, F(3, 81) = 0.564, MSE = 0.024, p = 0.640, ηp^2^ = 0.020. The grand average of all RTs was 1.327 s (95% CI = [1.175, 1.480]).

##### Deadline food-cueing experiment (DLFD)

**FIGURE 5:**
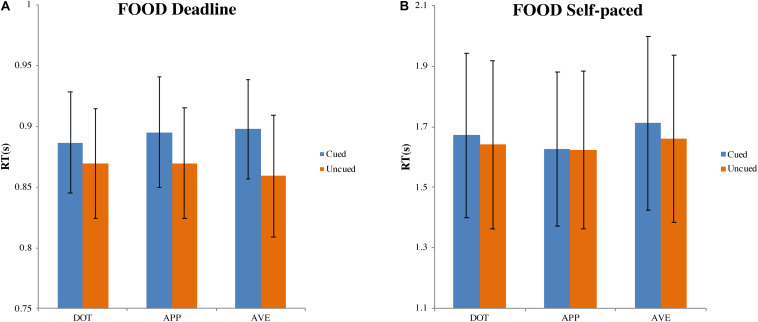
Mean response time and standard errors in the food-cueing experiments. **(A,B)** Represent the results of the deadline and the self-paced experiment respectively. Both panels show the data for different types of cue: dot (DOT), appetitive food images (APP), and aversive food images (AVE). For each type of cue, the blue bars pertain to the response times when choosing cued targets (Cued); the orange bars pertain to the response times when choosing uncued targets (Uncued). The error bars represent the 95% confidence interval around the mean in each condition.

In the DLFD (Figure 5A), we obtained a significant main effect of the type of choice on RT, F(1, 31) = 18.772, MSE = 0.002, p < 0.001, ηp^2^ = 0.377. There was no influence from the cue type on RT, F(1.472, 45.625) = 0.351, MSE = 0.002, p = 0.640, ηp^2^ = 0.011; nor was there an interaction between choice and cue, F(2, 62) = 2.221, MSE = 0.001, p = 0.117, ηp^2^ = 0.067. Compared with the mean RT for choosing the cued image (MCUED = 0.893, 95% CI = [0.851, 0.935]), the mean RT for choosing the uncued image was faster (MUNCUED = 0.866, 95% CI = [0.820, 0.913]).

##### Self-paced food-cueing experiment (SPFD)

In the SPFD (Figure 5B), there was no significant effect of choice, F(1, 31) = 1.938, MSE = 0.020, p = 0.174, ηp^2^ = 0.059, nor of the cue type, F(2, 62) = 3.235, MSE = 0.020, p = 0.054, ηp^2^ = 0.090. There was also no interaction between choice and cue type, F(2, 62) = 0.651, MSE = 0.015, p = 0.525, ηp^2^ = 0.021. The grand average of all RTs was 1.655 s (95% CI = [1.387, 1.924]).

### Gaze Analysis

For the gaze analysis, we followed the same procedure as in previous studies (Shimojo et al., 2003; Zommara et al., 2018) to plot the gaze distribution. In order to know where the subject’s gaze was located and how the gaze distribution changed, we conducted the analysis with two areas of interest: the left and right hemi-fields (i.e., half screen). We assigned a value of “1” if the subject’s gaze was directed to the same side of the cue; a value of “0” if the gaze was on the other side; and “not-a-number (NA)” if the gaze was outside of the screen. All gaze sampling points, from the onset of the pre-cue until the average response time in each experiment, were calculated to obtain the likelihood of looking at the cueing side, by averaging across all trials and subjects. Based on this analysis method, if the likelihood value is higher than “0.5,” it means there is a gaze bias toward the cueing side.

**FIGURE 6:**
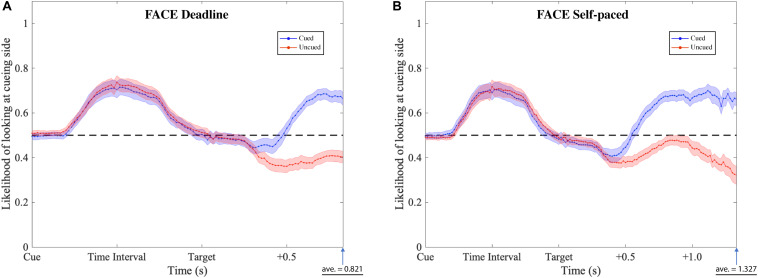
Gaze likelihood analysis of the face-cueing experiments. **(A,B)** Represent the result of the deadline and the self-paced experiment respectively. The likelihood that a subject’s gaze was directed toward the cueing side is plotted against the time past from the onset of the cue until the average response time. The black dashed lines reflect the chance level (0.5) of likelihood. The blue dotted lines represent the data when choosing the cued target (Cued); the red dotted lines represent the data when choosing the uncued target (Uncued). The shaded areas represent the range of the standard error of the mean for each plot.

**FIGURE 7:**
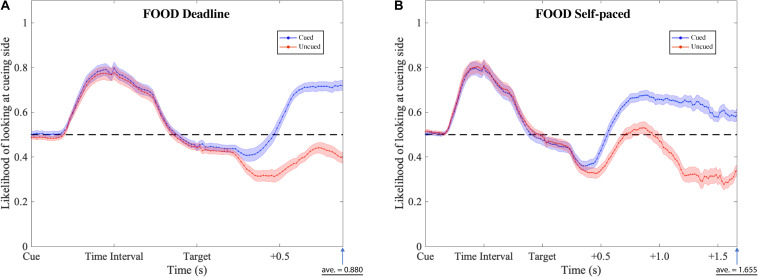
Gaze likelihood analysis of food-cueing experiments. **(A,B)** Represent the result of the deadline and the self-paced experiment respectively. The likelihood that a subject’s gaze was directed toward the cueing side is plotted against the time past from the onset of the cue until the average response time. The black dashed lines reflect the chance level (0.5) of likelihood. The blue dotted lines represent the data when choosing the cued target (Cued); the red dotted lines represent the data when choosing the uncued target (Uncued). The shaded areas represent the range of the standard error of the mean for each plot.

The gaze patterns were determined by the type of experiment (DLFC, SPFC, DLFD, and SPFD) and the ensuing choice (Cued versus Uncued), but there were no significant main effects or interactions with the affective value of the pre-cue. As presented in Figures 6, 7 (collapsed across types of pre-cue), the gaze curve showed similar patterns across all experiments. In all cases, the gaze likelihood at the onset of the pre-cue was at chance level (0.5), and then tended to shift toward the cueing side, peaking to a probability of around 0.7 for face images, and around 0.8 for food images. In order to establish the exact time of gaze shifting to the cueing side, we sub-divided the time into 20 bins of 50 ms (i.e., the time duration of cue and time interval). Two-tailed one-sample *t*-tests against 0.5 (for each bin, with Bonferroni correction) revealed a significant gaze bias, starting at 350 ms for choosing the cued image in DLFC, *p* < 0.001, starting at 300 ms for choosing the uncued image in DLFC, *p* < 0.001; and at 300 ms for both types of choice in SPFC, *p* < 0.001 (Figure 6). In the Food-cueing experiments, the gaze bias started at 300 ms for both choice conditions in DLFD, *p* < 0.001; and at 300 ms for choosing the cued image in SPFD, *p* < 0.001, versus at 250 ms for choosing the uncued image in SPFD, *p* < 0.001. In all cases, the gaze bias toward the cueing side ended, returning to chance level or even dipping below chance level, before the onset of the target screen.

Next, we investigated the differences between the two types of choice (cued versus uncued), by focusing on the data from the onset of the target screen. We sub-divided the time into 30 bins of 50 ms (i.e., 1.5 s from the onset of target screen). Paired-sample *t*-tests (for each bin, with Bonferroni correction) were performed comparing between the cued choices and the uncued choices. The results revealed significant differences, starting from 550 ms in the DLFC, *t*(27) = 4.200, *p* < 0.001, Cohen’s *d* = 0.794; and from 600 ms in the Self-paced Face-cueing experiment, *t*(27) = 3.846, *p* < 0.001, Cohen’s *d* = 0.727 (Figure 6). Significant differences were obtained starting from 450 ms in the Deadline Food-cueing experiment, *t*(31) = 3.928, *p* < 0.001, Cohen’s *d* = 0.694; and from 550 ms in the Self-paced Food-cueing experiment, *t*(31) = 4.547, *p* < 0.001, Cohen’s *d* = 0.804 (Figure 7). In all cases, the divergences reflected gaze biases toward the side of the ensuing choice.

Finally, in order to further investigate the start of the decision phase, we focused on the negative peak (i.e., lowest value below 0.5 in a 50 ms bin) in the data for the Cued choices at a time when the gaze curves between Cued and Uncued choices were not significantly different (i.e., before the divergence of the decision process, as determined above). From the hypothesis of IOR, we should expect this value to be significantly lower than 0.5. In line with accumulator models of preference formation, this would reflect a starting point bias toward the Uncued location in the decision process. For each of the four experiments, we therefore performed a two-tailed one-sample *t*-test against 0.5 for the pre-divergence negative peak in the Cued choices.

In the DLFC, the pre-divergence negative peak in the Cued choices was reached at 350 ms after Target onset (0.447). At this time there was no significant gaze bias in the direction of the Uncued location, *t*(27) = −1.905, *p* = 0.068, Cohen’s *d* = −0.360. In the Self-paced Face-cueing experiment, the peak was reached at 450 ms after Target onset (0.409). At this time there was a significant gaze bias in the direction of the Uncued location, *t*(27) = −3.115, *p* < 0.01, Cohen’s *d* = −0.589. Similar gaze biases in the direction of the Uncued location were obtained in the Deadline Food-cueing experiment, with a pre-divergence negative peak at 350 ms after Target onset (0.409), *t*(31) = −3.398, *p* < 0.005, Cohen’s *d* = −0.601; and in the Self-paced Food-cueing experiment, peaking at 400 ms (0.361), *t*(31) = −6.886, *p* < 0.001, Cohen’s *d* = −1.217.

## Discussion

The principal aim of this study was to elucidate the role of visual attention in the evaluative processing. To this end, we conducted four experiments to examine pre-cueing effects in a choice task based on perceived attractiveness. In our experimental paradigm, we varied both the type of cue (face versus food images, with different affective associations) and the time constraints (1.5 s deadline versus self-paced). The task for subjects was to choose the more preferred picture from a pair of abstract art images. We measured the choice probabilities, the response times, and the gaze patterns. Across the experiments we found a highly consistent pattern of results, with clear cueing effects on all three measures. First, with respect to the choice probabilities, there were significant influences from pre-cues with negative associations, but only under urgency. In deadline experiments, subjects tended to choose the uncued target, both following a disgusted face and following an aversive food image. No other choice biases were obtained. Second, with respect to the response times, choices for uncued targets tended to be faster than choices for cued targets, but only under urgency. Again, this pattern was consistent across experiments, both for face cues and for food cues. Finally, with respect to gaze likelihood, in all experiments, the pre-cues elicited a clear gaze bias to the cueing side, which subsequently dissipated before the onset of the target screen and further tended to develop into a bias in the opposite direction, in line with the phenomenon of IOR. From a starting point biased to the uncued location, the gaze patterns then diverged in the direction of the ensuing choice.

Taken together, the data supported the information-integration hypothesis, but not the gradual-commitment hypothesis. The affective value of the pre-cue had a decisive impact on the subsequent choice under urgency, indicating that the information was integrated in the preference formation. The gaze data and manual response times further suggested that the pre-cues had been effective, in agreement with the phenomenon of IOR. The cues elicited a biphasic gaze bias in opposite directions, first to the cued location and then to the opposite direction. Under urgency, the manual responses were faster for uncued targets than for cued targets.

### Cueing Effects on Preference Formation

In both deadline experiments, DLFC and DLFD, the results of negative cues (i.e., disgusted face and aversive food) indicated that the probability of choosing the uncued target was significantly greater than chance (0.573 in DLFC; 0.555 in DLFD). No significant difference was found when using other cues (i.e., neutral and positive cues). Additionally, the results in both deadline experiments showed that the response time when choosing uncued targets was faster than that when choosing cued targets.

The most plausible explanation, in line with a vast amount of research on covert visual attention, is that the pre-cues attracted not only overt attention, as evidenced by the gaze biases, but also covert attention, setting in motion the mechanisms that underlie IOR (engagement, disengagement, shift). This can explain why uncued targets were chosen faster than cued targets, regardless of the type of cue, in deadline experiments. Conversely, the fact that there were no response-time differences in the self-paced experiments suggests a short-lived influence of IOR. Such influence dissipated when subjects took more time to reflect on their preferred choice.

Although the attentional processes were activated by all pre-cues, the nature of the influence changed depending on the affective value of the pre-cue. Neutral cues in all experiments failed to influence the direction of the subsequent choice. In this sense, it appears that the phenomenon of IOR by itself does not suffice to influence the actual process of preference formation. Similarly, positive cues (happy face or appetitive food) had no measurable impact on the direction of choice. However, the clear choice bias following negative cues under urgency indicated that the affective value interacted with the attentional processing. Effectively, the value of the cued position had decreased, and thus impeded the selection of the target image that appeared there. We suggest that the negative affective value promoted attentional disengagement, leading to a pronounced bias against the same position. In other words, the negative value was integrated in the preference formation in such a way as to favor the alternate choice. At a more general level, this finding is consistent with a host of studies on the valence-dependent impact of emotion on attention (e.g., Tomkins, 1970; Toda, 1980; Lerner et al., 2015).

It should be noted that the preference formation here reflects a coalescence of object evaluation and manual choice behavior. In the present experiments, subjects were always required to select their preferred object. Consequently, we cannot establish whether the influence of affective cues under urgency are due to an actual increase in the evaluation of the chosen objects, or a more general preference to press a button opposite cues with negative associations. The critical test to disambiguate these possibilities would be to require subjects to pick the less preferred object. If, in such a test, the manual choice pattern switches, the influence of negative cues under urgency must pertain to the actual evaluation of the objects. Conversely, if the manual choice pattern stays the same, the effects would be due to response association at the level of manual motor control.

Interestingly, a study on consequences of attentional inhibition during visual search suggested that the spatial control of attention may have an impact on the actual evaluation of objects (Raymond et al., 2005). The paradigm consisted of search trials followed by evaluation trials. During search trials, subjects were required to locate colored patterns or tinted faces. In subsequent evaluation trials, subjects were required to evaluate objects that had been either distractors or targets in the search trials. Distractors were rated lower than targets, and this effect was more pronounced when the distractors had been near the targets during the search trials. The effect of target-distractor proximity was observed even when the objects were presented at different locations in the evaluation trials. Overall, the data suggested that location-based suppression translated into emotional devaluation. Future work can build on this paradigm, as well as ours in the present study, to examine the exact nature of the influences of attentional control on preference formation.

### The Role of Urgency

By comparing the results of deadline experiments and self-paced experiments, we aimed to elucidate the impact of urgency, both on evaluative decision-making as a whole, and specifically on the pattern of cueing effects. To be sure, our results confirmed that, compared with self-paced experiments, deadline experiments yielded much faster response times (an overall difference of more than 500 ms in face-cue experiments, and more than 700 ms in food-cue experiments). The gaze-likelihood analyses also showed earlier divergence toward the preferred side in deadline experiments as compared to self-paced experiments. Importantly, in self-paced experiments we found no significant effects at all from pre-cues on the manual responses. When subjects were free to take as much time as they wanted, there were traces of IOR in the gaze patterns, with gaze biases that clearly showed a biphasic process of moving toward, and then away from, the cued location, but this did not translate into affective influences in the response times and choice probabilities. In terms of accumulator models (Krajbich et al., 2010; Gluth et al., 2018, 2020), the results imply that the pre-cues produced a bias in the starting position of the evidence accumulation, but, with respect to preference formation, the pre-cues mattered only under urgency.

Urgency effectively changes the threshold for decision-making (Reddi and Carpenter, 2000; Noorani, 2014; Noorani and Carpenter, 2016). When given less time for choosing, people must take shortcuts in the decision-making process. Reddi and Carpenter (2000), adopting the LATER model for decision-making (Carpenter, 1981), pointed out that the accelerative effect of urgency is mainly attributable to its influence on the threshold at which a response is initiated, not the efficiency of information processing. Subjects would lower their decision threshold under urgency, while still extracting information as efficiently as they can. This implies that pre-existing response biases have an exacerbated effect under urgency. Accordingly, with response time shortened and the efficiency of information processing unvaried, it is reasonable to assume that, in the present study, subjects acquired less information from the targets in the deadline experiments than in the self-paced experiments. We suspect that the gap in the amount of information available to the subjects in deadline versus self-paced experiments may explain why the cues were effective only under urgency. The cueing, as one form of information, became relatively more significant as the total amount of information decreased, and could therefore have a greater impact on the evaluative decision-making under urgency. In other words, the cueing effects may be enhanced when less information is available to the subjects, and weakened otherwise.

### Additive Versus Multiplicative Role of Attention in Preference Formation

In previous studies, the gaze cascade phenomenon has been demonstrated multiple times with a range of variations, including both preferential tasks (Shimojo et al., 2003; Simion and Shimojo, 2006, 2007; Bird et al., 2012) and non-preferential tasks (Glaholt and Reingold, 2009; Nittono and Wada, 2009; Schotter et al., 2010; Morii and Sakagami, 2015; Zommara et al., 2018). Shimojo et al. (2003) interpreted the phenomenon as an active positive influence from the gaze on preference formation (i.e., the gradual-commitment hypothesis). Phrased in terms of accumulator models (Krajbich et al., 2010; Gluth et al., 2018, 2020), the gradual-commitment hypothesis implies that gaze duration leads to a positive additive effect on preference formation. In the present study, we obtained significant gaze biases toward the ensuing choices in all conditions. However, the present cueing effects under urgency appear in conflict with the notion of a positive additive effect from the gaze on preference formation. Despite the fact that the gaze tended to go to the pre-cues in all conditions, this did not lead to any advantage in the preference formation for the cueing side. Moreover, the negative influences from the face cue with an expression of disgust and from aversive-food cues cannot easily be reconciled with the notion that viewing leads to liking.

Instead, we propose that the gaze, as a form of overt attention, can overlap with covert attention to show IOR and to perform a function of information integration. The integrated information can either promote or impede preference formation depending on its valence. In terms of accumulator models of preference formation, the information-integration hypothesis appears consistent with a multiplicative role of attention, as often touted in neurophysiological investigations of attention (e.g., Stoppel et al., 2011; Lee et al., 2016). Multiplicative gain modulation by attention might explain how gazing at negatively valued objects leads to further devaluation of the attended objects. Nevertheless, we cannot rule out a form of scaling that leads to subtraction of value through additive inhibitory mechanisms (Ayaz and Chance, 2009), or more complex forms of information integration with sequential stages of multiplicative, and then additive or subtractive, scaling (Andersen et al., 2012). While it may be difficult to actually model the influence of attention on preference formation in the present paradigm, it seems appropriate at least to rule out the notion of “Pavlovian approach.” There is not necessarily a positive additive effect from attention on preference formation.

This interpretation mirrors other findings in our own laboratory, undercutting the notion that viewing leads to liking. Using an absolute-evaluation paradigm with food images, we found that in most situations, viewing time was not associated with increased preference formation when subjects evaluated food images one at a time, rather than making choices between pairs of images (Wolf et al., 2018, 2019). The entire pattern of data suggested that increased viewing times occurred under indecision or deliberative processing, that is, when subjects engaged in a prolonged effort of information integration. Conversely, the phenomenon of the gaze cascade may depend on the decision goal (Van der Laan et al., 2015), particularly in situations where the gaze can act as a spatial precursor to choice (Gerbella et al., 2017). In other words, the gaze would not have a direct positive impact on preference formation at all, but might perform a role in guiding motor behavior toward a spatial choice. The latter function would create the impression that viewing leads to liking in situations where liking is expressed as a spatial choice.

## Data Availability Statement

All datasets generated for this study are included in the article/Supplementary Material.

## Ethics Statement

The present study was approved by the Human Ethics Committee of the Faculty of Arts and Science, Kyushu University (Issue No. 201801). Informed consent was obtained in writing from each subject. The patients/participants provided their written informed consent to participate in this study.

## Author Contributions

JX conducted the data collection for the study, analyzed the behavior and eye-tracking data, and prepared all the figures. JX, NZ, and KO programmed the experiments. JX and JL wrote the manuscript. All authors contributed to the design of the study and reviewed and approved the manuscript.

## Conflict of Interest

The authors declare that the research was conducted in the absence of any commercial or financial relationships that could be construed as a potential conflict of interest.

## Supplementary Material

DATA SHEET S1 Complete data set for manual responses in the present study, organized in four Data Sheets: Choice_Probability_FACE; Choice_Probability_FOOD; RT_FACE; and RT_FOOD.

DATA SHEET S2 Complete data set of gaze likelihood in the present study, organized in four Data Sheets: FACE_Deadline; FACE_Self-paced; FOOD_Deadline; and FOOD_Self-paced.

## References

[B1] Andersen S. K. Müller M. M. Martinovic J. (2012). Bottom-up biases in feature-selective attention. *J. Neurosci.* 32 16953–16958. 10.1523/JNEUROSCI.1762-12.2012 23175846 PMC6621795

[B2] Armaghani S. J. Crucian G. P. Heilman K. M. (2014). The influence of emotional faces on the spatial allocation of attention. *Brain Cogn.* 91 108–112. 10.1016/j.bandc.2014.09.006 25306560

[B3] Atkinson M. A. Simpson A. A. Cole G. G. (2018). Visual attention and action: how cueing, direct mapping, and social interactions drive orienting. *Psychon. Bull. Rev.* 25 1585–1605. 10.3758/s13423-017-1354-0 28808932

[B4] Ayaz A. Chance F. S. (2009). Gain modulation of neuronal responses by subtractive and divisive mechanisms of inhibition. *J. Neurophysiol.* 101 958–968. 10.1152/jn.90547.2008 19073814

[B5] Bird G. D. Lauwereyns J. Crawford M. T. (2012). The role of eye movements in decision making and the prospect of exposure effects. *Vis. Res.* 60 16–21. 10.1016/j.visres.2012.02.014 22425778

[B6] Carpenter R. H. S. (1981). “Oculomotor procrastination,” in *Eye Movements: Cognition and Visual Perception*, eds Fisher D. F. Monty R. A. Senders J. W. (Hillsdale: Lawrence Erlbaum), 237–246. 10.4324/9781315437415-19

[B7] Carrasco M. (2011). Visual attention: the past 25 years. *Vis. Res.* 51 1484–1525. 10.1016/j.visres.2011.04.012 21549742 PMC3390154

[B8] Denefrio S. Simmons A. Jha A. Dennis-Tiwary T. A. (2017). Emotional cue validity effects: the role of neurocognitive responses to emotion. *PLoS One* 12:e0179714. 10.1371/journal.pone.0179714 28683069 PMC5499989

[B9] Foroni F. Pergola G. Argiris G. Rumiati R. I. (2013). The FoodCast research image database (FRIDa). *Front. Hum. Neurosci.* 7:51. 10.3389/fnhum.2013.00051 23459781 PMC3585434

[B10] Fox E. Russo R. Dutton K. (2002). Attentional bias for threat: evidence for delayed disengagement from emotional faces. *Cogn. Emot.* 16 355–379. 10.1080/02699930143000527 18273395 PMC2241753

[B11] Gerbella M. Rozzi S. Rizzolatti G. (2017). The extended object-grasping network. *Exp. Brain Res.* 235 2903–2916. 10.1007/s00221-017-5007-3 28748312

[B12] Glaholt M. G. Reingold E. M. (2009). Stimulus exposure and gaze bias: a further test of the gaze cascade model. *Atten. Percept. Psychophys.* 71 445–450. 10.3758/APP.71.3.445 19304635

[B13] Gluth S. Kern N. Kortmann M. Vitali C. L. (2020). Value-based attention but not divisive normalization influences decisions with multiple alternatives. *Nat. Hum. Behav.* 10.1038/s41562-020-0822-0 [Online ahead of print] 32015490 PMC7306407

[B14] Gluth S. Spektor M. S. Rieskamp J. (2018). Value-based attentional capture affects multi-alternative decision making. *eLife* 7:e39659. 10.7554/eLife.39659 30394874 PMC6218187

[B15] Handy T. C. Jha A. P. Mangun G. R. (1999). Promoting novelty in vision: inhibition of return modulates perceptual-level processing. *Psychol. Sci.* 10 157–161. 10.1111/1467-9280.00124

[B16] Klein R. (1988). Inhibitory tagging system facilitates visual search. *Nature* 334 430–431. 10.1038/334430a0 3405288

[B17] Klein R. M. (2000). Inhibition of return. *Trends Cogn. Sci.* 4 138–147. 10.1016/S1364-6613(00)01452-2 10740278

[B18] Krajbich I. Armel C. Rangel A. (2010). Visual fixations and the computation and comparison of value in simple choice. *Nat. Neurosci.* 13 1292–1298. 10.1038/nn.2635 20835253

[B19] Kunar M. A. Watson D. G. Tsetsos K. Chater N. (2017). The influence of attention on value integration. *Atten. Percept. Psychophys.* 79 1615–1627. 10.3758/s13414-017-1340-7 28547680 PMC5515995

[B20] Lauwereyns J. (2012). *Brain and the Gaze: On the Active Boundaries of Vision.* Cambridge, MA: The MIT Press.

[B21] Lee C. C. Diamond M. E. Arabzadeh E. (2016). Sensory prioritization in rats: behavioral performance and neuronal correlates. *J. Neurosci.* 36 3243–3253. 10.1523/JNEUROSCI.3636-15.2016 26985034 PMC6705526

[B22] Lerner J. Li Y. Valdesolo P. Kassam K. (2015). Emotion and decision making. *Annu. Rev. Psychol.* 66 799–823. 10.1146/annurev-psych-010213-115043 25251484

[B23] Li Y. Long J. Huang B. Yu T. Wu W. Li P. (2016). Selective audiovisual semantic integration enabled by feature-selective attention. *Sci. Rep.* 6:18914. 10.1038/srep18914 26759193 PMC4725371

[B24] Mathews A. Mackintosh B. Fulcher E. P. (1997). Cognitive biases in anxiety and attention to threat. *Trends Cogn. Sci.* 1 340–345. 10.1016/S1364-6613(97)01092-9 21223944

[B25] Morii M. Sakagami T. (2015). The effect of gaze-contingent stimulus elimination on preference judgments. *Front. Psychol.* 6:1351. 10.3389/fpsyg.2015.01351 26441727 PMC4563161

[B26] Nittono H. Wada Y. (2009). Gaze shifts do not affect preference judgments of graphic patterns. *Percept. Mot. Skills* 109 79–94. 10.2466/PMS.109.1.79-94 19831089

[B27] Noorani I. (2014). LATER models of neural decision behavior in choice tasks. *Front. Integr. Neurosci.* 8:67. 10.3389/fnint.2014.00067 25202242 PMC4141543

[B28] Noorani I. Carpenter R. H. S. (2016). The LATER model of reaction time and decision. *Neurosci. Biobehav. Rev.* 64 229–251. 10.1016/j.neubiorev.2016.02.018 26915927

[B29] Ooms K. Dupont L. Lapon L. Popelka S. (2015). Accuracy and precision of fixation locations recorded with the low-cost Eye Tribe tracker in different experimental set - ups. *J. Eye Mov. Res.* 8 1–24. 10.16910/jemr.8.1.5

[B30] Ounjai K. Kobayashi S. Takahashi M. Matsuda T. Lauwereyns J. (2018). Active confirmation bias in the evaluative processing of food images. *Sci. Rep.* 8:16864. 10.1038/s41598-018-35179-9 30443034 PMC6237889

[B31] Peirce J. Gray J. R. Simpson S. MacAskill M. Höchenberger R. Sogo H. (2019). PsychoPy2: experiments in behavior made easy. *Behav. Res. Methods* 51 195–203. 10.3758/s13428-018-01193-y 30734206 PMC6420413

[B32] Peirce J. W. (2009). Generating stimuli for neuroscience using psychopy. *Front. Neuroinformatics* 2:8. 10.3389/neuro.11.010.2008 19198666 PMC2636899

[B33] Pool E. R. Pauli W. M. Kress C. S. O’Doherty J. P. (2019). Behavioural evidence for parallel outcome-sensitive and outcome-insensitive Pavlovian learning systems in humans. *Nat. Hum. Behav.* 3 284–296. 10.1038/s41562-018-0527-9 30882043 PMC6416744

[B34] Posner M. I. (1980). Orienting of attention. *Q. J. Exp. Psychol.* 32 3–25. 10.1080/00335558008248231 7367577

[B35] Posner M. I. Cohen Y. (1984). “Components of Visual Orienting,” in *Attention and Performance X*, eds Bouma H. Bowhuis D. (Hillsdale, NJ: Erlbaum), 531–556.

[B36] Pratt J. Kingstone A. Khoe W. (1997). Inhibition of return in location- and identity-based choice decision tasks. *Percept. Psychophys.* 59 964–971. 10.3758/bf03205511 9270368

[B37] Rastegary H. Landy F. J. (1993). “The interactions among time urgency, uncertainty, and time pressure,” in *Time Pressure and Stress in Human Judgment and Decision Making*, eds Svenson O. Maule A. J. (Boston, MA: Springer US), 217–239. 10.1007/978-1-4757-6846-6_15

[B38] Raymond J. E. Fenske M. J. Westoby N. (2005). Emotional devaluation of distracting patterns and faces: a consequence of attentional inhibition during visual Search? *J. Exp. Psychol.: Hum. Percept. Perform.* 31 1404–1415. 10.1037/0096-1523.31.6.1404 16366798

[B39] Reddi B. A. Carpenter R. H. S. (2000). The influence of urgency on decision time. *Nat. Neurosci.* 3 827–830. 10.1038/77739 10903577

[B40] Sakai K. Miyashita Y. (1991). Neural organization for the long-term memory of paired associates. *Nature* 354 152–155. 10.1038/354152a0 1944594

[B41] Scharinger M. Steinberg J. Tavano A. (2017). Integrating speech in time depends on temporal expectancies and attention. *Cortex* 93 28–40. 10.1016/j.cortex.2017.05.001 28609683

[B42] Schotter E. R. Berry R. W. McKenzie C. R. M. Rayner K. (2010). Gaze bias: selective encoding and liking effects. *Vis. Cogn.* 18 1113–1132. 10.1080/13506281003668900

[B43] Shimojo S. Simion C. Shimojo E. Scheier C. (2003). Gaze bias both reflects and influences preference. *Nat. Neurosci.* 6 1317–1322. 10.1038/nn1150 14608360

[B44] Simion C. Shimojo S. (2006). Early interactions between orienting, visual sampling and decision making in facial preference. *Vis. Res.* 46 3331–3335. 10.1016/j.visres.2006.04.019 16765404

[B45] Simion C. Shimojo S. (2007). Interrupting the cascade: orienting contributes to decision making even in the absence of visual stimulation. *Percept. Psychophys.* 69 591–595. 10.3758/BF03193916 17727112

[B46] Stevens J. (2008). “Stevens, J.R. (2008) The evolutionary biology of decision making,” in *Better than Conscious? Decision Making, the Human Mind, and Implications for Institutions*, eds Engel C. Singer W. (Cambridge, MA: MIT Press), 285–304. 10.7551/mitpress/9780262195805.003.0013

[B47] Stoppel C. M. Boehler C. N. Strumpf H. Heinze H.-J. Noesselt T. Hopf J.-M. (2011). Feature-based attention modulates direction-selective hemodynamic activity within human MT. *Hum. Brain Mapp.* 32 2183–2192. 10.1002/hbm.21180 21305663 PMC6870266

[B48] Stoyanova R. S. Pratt J. Anderson A. K. (2007). Inhibition of return to social signals of fear. *Emot. Wash. DC* 7 49–56. 10.1037/1528-3542.7.1.49 17352562

[B49] Suri R. Monroe K. B. (2003). The effects of time constraints on consumers’ judgments of prices and products. *J. Consum. Res.* 30 92–104. 10.1086/374696

[B50] Taylor T. L. Therrien M. E. (2005). Inhibition of return for faces. *Percept. Psychophys.* 67 1414–1422. 10.3758/bf03193646 16555593

[B51] Theeuwes J. Van der Stigchel S. (2006). Faces capture attention: evidence from inhibition of return. *Vis. Cogn.* 13 657–665. 10.1080/13506280500410949

[B52] Tipper S. P. Weaver B. Jerreat L. M. Burak A. L. (1994). Object-based and environment-based inhibition of return of visual attention. *J. Exp. Psychol. Hum. Percept. Perform.* 20 478–499. 10.1037/0096-1523.20.3.478 8027711

[B53] Toda M. (1980). Emotion and decision making. *Acta Psychol.* 45 133–155. 10.1016/0001-6918(80)90026-8

[B54] Tomkins S. (1970). “Affects as the Primary Motivational System,” in *Feelings and Emotions*, ed. Arnold M. B. (New York, NY: Academic Press), 101–110. 10.1016/b978-0-12-063550-4.50013-9

[B55] Tottenham N. Tanaka J. W. Leon A. C. McCarry T. Nurse M. Hare T. A. (2009). The NimStim set of facial expressions: judgments from untrained research participants. *Psychiatry Res.* 168 242–249. 10.1016/j.psychres.2008.05.006 19564050 PMC3474329

[B56] Treisman A. M. Gelade G. (1980). A feature-integration theory of attention. *Cognit. Psychol.* 12 97–136. 10.1016/0010-0285(80)90005-5 7351125

[B57] Van der Laan L. N. Hooge I. T. C. de Ridder D. T. D. Viergever M. A. Smeets P. A. M. (2015). Do you like what you see? The role of first fixation and total fixation duration in consumer choice. *Food Qual. Prefer.* 39 46–55. 10.1016/j.foodqual.2014.06.015

[B58] Vuilleumier P. Schwartz S. (2001). Emotional facial expressions capture attention. *Neurology* 56 153–158. 10.1212/wnl.56.2.153 11160948

[B59] Waller M. J. Conte J. M. Gibson C. B. Carpenter M. A. (2001). The effect of individual perceptions of deadlines on teamperformance. *Acad. Manage. Rev.* 26 586–600. 10.2307/3560243

[B60] Weaver M. D. Aronsen D. Lauwereyns J. (2012). A short-lived face alert during inhibition of return. *Atten. Percept. Psychophys.* 74 510–520. 10.3758/s13414-011-0258-8 22205613

[B61] Wolf A. Ounjai K. Takahashi M. Kobayashi S. Matsuda T. Lauwereyns J. (2018). Evaluative processing of food images: a conditional role for viewing in preference formation. *Front. Psychol.* 9:936. 10.3389/fpsyg.2018.00936 29942273 PMC6004500

[B62] Wolf A. Ounjai K. Takahashi M. Kobayashi S. Matsuda T. Lauwereyns J. (2019). Evaluative processing of food images: longer viewing for indecisive preference formation. *Front. Psychol.* 10:608. 10.3389/fpsyg.2019.00608 30949106 PMC6435591

[B63] Xu J. Zommara N. Lauwereyns J. (2016). “The role of visual attention in preference formation for food,” in *Proceedings of the Official Conference Proceedings of the Asian Conference on Psychology and the Behavioral Sciences* (Nagoya: The International Academic Forum), 209–220.

[B64] Zommara N. M. Takahashi M. Ounjai K. Lauwereyns J. (2018). A gaze bias with coarse spatial indexing during a gambling task. *Cogn. Neurodyn.* 12 171–181. 10.1007/s11571-017-9463-z 29564026 PMC5852011

